# Facile Synthesis of Microporous Carbons from Biomass Waste as High Performance Supports for Dehydrogenation of Formic Acid

**DOI:** 10.3390/nano11113028

**Published:** 2021-11-11

**Authors:** Tingting Cao, Jinke Cheng, Jun Ma, Chunliang Yang, Mengqin Yao, Fei Liu, Min Deng, Xiaodan Wang, Yuan Ren

**Affiliations:** 1 School of Chemistry and Chemical Engineering, Guizhou University, Guiyang 550025, China; caotingting980417@163.com (T.C.); jkcheng@gzu.edu.cn (J.C.); jma3@gzu.edu.cn (J.M.); clyang@gzu.edu.cn (C.Y.); gzu403299473@163.com (M.D.); wangxiaodan0516@126.com (X.W.); 2 Key Laboratory of Green Chemical and Clean Energy Technology, Guizhou University, Guiyang 550025, China

**Keywords:** porous carbon, Pd nano-catalysts, agriculture waste, formic acid, dehydrogenation

## Abstract

Formic acid (FA) is found to be a potential candidate for the storage of hydrogen. For dehydrogenation of FA, the supports of our catalysts were acquired by conducting ZnCl_2_ treatment and carbonation for biomass waste. The texture and surface properties significantly affected the size and dispersion of Pd and its interaction with the support so as to cause the superior catalytic performance of catalysts. Microporous carbon obtained by carbonization of ZnCl_2_ activated peanut shells (C_PS_-ZnCl_2_) possessing surface areas of 629 m^2^·g^−1^ and a micropore rate of 73.5%. For ZnCl_2_ activated melon seed (C_MS_-ZnCl_2_), the surface area and micropore rate increased to 1081 m^2^·g^−1^ and 80.0%, respectively. In addition, the introduction of ZnCl_2_ also caused the increase in surface O content and reduced the acidity of the catalyst. The results represented that C_MS_-ZnCl_2_ with uniform honeycomb morphology displayed the best properties, and the as-prepared Pd/C_MS_-ZnCl_2_ catalyst afforded 100% hydrogen selectivity as well as excellent catalytic activity with an initial high turnover number (TON) value of 28.3 at 30 °C and 100.1 at 60 °C.

## 1. Introduction

With the rapid development of social science and technology, a large amount of traditional fossil energy is consumed in the process of industrialization. The consumption of fossil energy has produced a series of problems such as energy crisis and environmental pollution, which makes human society put forward an urgent demand for a new energy system [1,2]. Hydrogen, with the major advantages of cleanness, high efficiency, high energy density (142 MJ·kg^−1^) and renewability, is one of the most promising energy carriers [3]. However, the preparation, storage, transportation and conversion of hydrogen are the main obstacles to the practical application of hydrogen [4,5]. At present, liquid phase hydrogen storage materials, which store hydrogen energy in the form of chemical bonds and produce hydrogen by catalytic dehydrogenation, are considered to be safe and efficient candidate materials [6,7]. Among them, hydrogen mass density and volume capacity of formic acid (FA) are as high as 4.4 wt% and 53.4 g·L^−1^, respectively. It is a non-toxic, harmless and cheap chemical hydrogen storage material that can effectively avoid the problem of hydrogen storage and transportation [8,9]. Through a cycle constituted of dehydrogenation of FA and CO_2_ hydrogenation, C1 resources can be effectively recycled, and zero carbon dioxide emission can be realized in theory, which is of great significance in the field of energy and environment [10,11]. Under appropriate catalyst and reaction conditions, FA can be decomposed either through dehydrogenation (Equation (1)) or dehydration (Equation (2)). Obviously, the key to promote the practical application of FA as hydrogen storage material is to design and synthesize highly active and selective catalysts to decompose FA to hydrogen through dehydrogenation reaction.

(1) $$\mathrm{HCOOH}\to H_{2}+{\mathrm{CO}}_{2}$$

(2) $$\mathrm{HCOOH}\to H_{2}O+\mathrm{CO}$$

A large number of research results showed that almost all heterogeneous catalysts effective for the decomposition of FA to hydrogen contain metal Pd, yet their catalytic activity and selectivity still cannot meet the requirements of practical application [12,13]. By introducing another metal to form Pd-based alloy catalysts, such as Pd-Au and Pd-Ag, the adsorption force of Pd on FA could be adjusted to promote dehydrogenation efficiency [14]. In addition, metal nanoparticles with small particle size usually show superior catalytic performance than large nanoparticles in FA dehydrogenation. Typically, capping agents are used in the preparation of small metal particles to resist thermodynamic instability due to their high surface energy [15,16]. However, capping molecules will pollute the active sites and result in the loss of catalytic activity. Therefore, anchoring active metal nanoparticles on suitable supports is an effective strategy for producing stable small particles and ensuring optimal catalyst utilization [17,18,19]. Porous carbon materials are ideal supports on account of its large surface area, well-developed pore structure and acid and alkali resistant properties [20,21]. Masuda et al. reported that Pd nanoparticles supported commercial Maxsorb carbon MSC-30, which had a high turnover frequency value for Pd/MSC-30 of FA decomposition that reached 5638 h^−1^ at 75 °C [22]. Lee et al. prepared hierarchically porous graphene-like carbon (KIE-8) support by chitosan, urea and KOH mixture pyrolysis methods, and the resulting catalyst for FA decomposition was 287 h^−1^ at room temperature [23].

The natural biomass material is rich in carbon elements, renewable, easy to process and is low cost as a potential carbon material precursor [24,25,26]. However, the pore structure of carbon materials prepared by direct pyrolysis of biomass precursors is generally underdeveloped and mostly has block structures and single chemical compositions, so it is not suitable for use as support materials [27,28]. Therefore, the activation of biomass carbon materials is an auxiliary but essential strategy for improving the material’s chemical state, internal 3D network and nanostructures [29]. Promoters such as sodium hydroxide/potassium (KOH/NaOH), potassium chloride (KCl), potassium carbonate (K_2_CO_3_), zinc chloride (ZnCl_2_) and phosphoric acid (H_3_PO_4_) are usually mixed with biomass precursors to form porous structures. The basic mechanism is that the surface carbon of biomass can be consumed by activators at high temperature so as to introduce layered porosity [30]. Nuilek et al. reported that the specific surface area of biomass carbon could be improved by activating Urtica with KOH [31]. With H_3_PO_4_ activation of xylan, the specific surface area of carbons increased from 548 to 1558 m^2^·g^−1^ [32].

According to information compiled by China Rural Statistical Yearbook, the total peanut and melon seed productions were estimated at about 17.52 million tons and 26.64 million tons in 2019 [33]. If the shell accounts for 30% of the total weight, about 6 million tons of peanut shell and 9 million tons of melon seed shell can be produced. As by-products, most of them are discarded or burned, except for a few that are used in processing animal feed and edible fungus culture medium, resulting in a serious waste of resources and environmental pollution [34,35]. Using them as precursor of biomass carbon is a win-win strategy, which not only can realize resource utilization of agricultural waste but also achieve the goals of carbon neutrality and peak carbon dioxide emissions. Herein, a series of biomass carbon supports was prepared by pyrolysis of peanut shell and melon seed shell to synthesize Pd/C catalyzed dehydrogenation of FA. The influence of the support structure and surface properties on the catalytic performance of the catalyst was discussed. As a result, the melon seed shell activated by ZnCl_2_ was used as the best support, and it is supported Pd nanoparticles possessing high activity and stability for the decomposition of FA.

## 2. Materials and Methods

### 2.1. Materials

Peanut seeds and melon seeds (the supermarket in Guiyang, China), formic acid (FA, HCOOH, Aladdin, AR, 98 vol%, Shanghai, China), sodium formate (SF, HCOONa, Aladdin, AR, 99.5 wt%, Shanghai, China), sodium borohydride (NaBH_4_, Acros, AR, 98 wt%, Shanghai, China), zinc chloride (ZnCl_2_, Macklin, GR, 99.8 wt%, Shanghai, China), nitric acid (HNO_3_, Chengdu Kelong, AR, 65–68 vol%, Chengdu, China), hydrochloric acid (HCl, Chengdu Kelong, AR, 36–38 vol%) and palladium chloride (PdCl_4_, Guiyan Platinum Industry, AR, 59.5 wt%, Guiyang, China) were not further purified. Ordinary deionized water was used as the reaction solvent.

### 2.2. Preparation of Catalysts

The peanut seed shell and melon shell were dried in an oven at 150 °C for 3 h, and their water contents measured by moisture meter were 10.28% and 10.00%, respectively. The dried biomasses were crushed by a crusher and sieved through 20 mesh. The samples were treated with a 50.0 vol% aqueous HNO_3_ solution for 30 min and then washed with deionized water to neutral drying for standby. The treated biomasses were dispersed in a ZnCl_2_ solution (the mass ratio of ZnCl_2_ to biomass was 2) and stirred at room temperature for 24 h. After drying, the moisture contents of peanut seed shell and melon shell treated with ZnCl_2_ were 9.72% and 9.57%, respectively. Biomass carbon was obtained by slow pyrolysis of biomass in a tubular furnace oven under continuous 200.0 mL·min^−1^ N_2_ flow at 800 °C (heating rate of 5 °C·min^−1^) for 2 h. The obtained black carbonized samples were washed to neutral with a 0.5 vol% aqueous HCl solution and labeled as C_PS_-ZnCl_2_ and C_MS_-ZnCl_2_ after drying. The acquisition of C_PS_ and C_MS_ followed the above steps except ZnCl_2_ activation.

H_2_PdCl_4_ was dropped into the support dispersed with deionized water by an incipient wetness method at 30 °C and stood for 24 h. Finally, the solids were reduced at a 0.1 mol·L^−1^ NaBH_4_ solution and dried at 80 °C vacuum oven for 2 h, and the reduced catalysts were marked as Pd/C_PS_ and Pd/C_MS_ and Pd/C_PS_-ZnCl_2_ and Pd/C_MS_-ZnCl_2_, in which the loads of Pd were 5 wt%.

### 2.3. Test of Catalysts

The decomposition of FA reaction was conducted in a three-necked flask (Figure S1). One neck of the reaction flask was reserved for introducing 10.0 mL of mixed solution of 1.0 mol·L^−1^ FA and 1.0 mol·L^−1^ SF, and another was connected to a gas burette. The third one was inserted into a thermocouple to measure the reaction temperature. After the mixed solution was injected to the reactor containing catalyst (n_Pd_/n_FA_ = 0.2%), the volume of the produced gas was recorded by recording the displacement of water in the gas burette. Unless otherwise specified, the reaction was carried out at 30 °C.

The used catalyst was recovered from the reaction mixture by filtration and then continuously washed with deionized water. After drying, an FA/SF solution was added for the recycling experiment.

### 2.4. Calculation Method

The activity of catalysts was compared and discussed in terms of turnover number and (TON) calculated according Equation (3).

(3) TON =PV2nPdRT

P denotes atmospheric pressure (101325 Pa), V denotes the generated volume of gas, n_Pd_ denotes the total mole number of Pd atoms in the catalyst, R denotes the universal gas constant (8.3145 m^3^·Pa·mol^−1^·K^−1^) and T denotes 25 °C (298 K). TON_X_ denotes TON at X min.

### 2.5. Characterization of Catalysts

The moisture content of biomass was obtained by a moisture meter (SFY-20W, Shenzhen, China). Biomasses were detected using a NETZSCH thermogravimetry (TG) 209 instrument (Billerica, MA, USA) with a ramp rate of 5 °C·min^−1^. X-ray diffraction (XRD) analysis was recorded on a Bruker D8 diffractometer (Billerica, MA, USA, 40 kV and 30 mA) using Cu irradiation (λ = 1.54178 Å). The structural characterization of the catalysts was measured by transmission electron microscopy (TEM) using a Phillips Analytical FEI Talos-S (Tokyo, Japan) operated at 200 kV. The surface area and pore volume were performed by means of N_2_ adsorption/desorption at −196 °C, after dehydration under vacuum at 180 °C for 6 h using ASAP 2460 (Norcross, GA, USA). According to the Brunauer–Emmett–Teller (BET) model, Barrett–Joyner–Halenda (BJH) method and the t-plot method, the surface areas, mesoporous volume and microporous volume were estimated, respectively. Scanning electron microscopy (SEM, ZEISS ΣIGMA, Tokyo, Japan) was used to observe the surface morphology of the catalysts. The contact angles of the catalysts were determined by using a Drop Shape Analysis System (Kruss DSA-100, Hamburg, Germany). Fourier Transform infrared spectroscopy (FTIR, IRAffinity-15, Shimane, Japan) was applied to record the absorption spectra of the catalysts. X-ray photoelectron spectroscopy (XPS) was carried out on a Fisher K-Alpha (Waltham, MA, USA) with monochromatic Al Kα (1486.7 eV) as an X-ray source. The metal content was determined by inductively coupled plasma optical emission spectroscopy (ICP-OES, Agilent ICPOES730, Santa Clara, California, U.S.). The reactive products generated from FA were determined by a GC-9560 (Shimane, Japan) with TCD detector (TDX-01 column) and hydrogen flame ionization detector (FID, Porapak-Q col-umn, Zurich, Switzerland).

## 3. Results and Discussion

### 3.1. Catalyst Characterization Results

**Figure 1:**
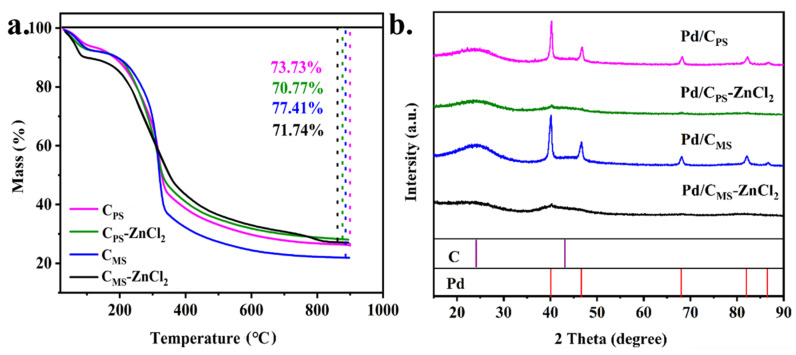
(**a**) TG curves of various supports and (**b**) XRD pattern of various catalysts.

TG analysis was used to explore the changes of biomasses during pyrolysis. As shown in Figure 1a, weight loss in the first stage occurred at about 110 °C, which was due to water evaporation, and about 10% of weight loss was also consistent with the water content in biomasses. The obvious weight loss in the second stage appeared at 200–500 °C, which might be caused by hemicellulose, cellulose and lignin decomposition [36,37]. When the temperature raised to 800 °C, weight loss of all samples tended to be stable, and weight loss rates were about 75%. The XRD pattern of the catalyst is shown in Figure 1b. These catalysts had a broad peak at about 2θ = 24° and 44°, corresponding to the (002) and (100) planes of carbon (PDF#01-0604). The diffraction peaks of Pd/C_PS_ and Pd/C_MS_ at 40.0°, 46.7° and 68.1° belonged to (111), (200) and (220) planes of Pd, respectively (PDF#01-1310). In Table 1, the average particle sizes of Pd/C_PS_ and Pd/C_MS_ calculated by the Scherrer equation (Equation (S1)) were 7.2 and 6.8 nm, while Pd/C_PS_-ZnCl_2_ and Pd/C_MS_-ZnCl_2_ with smaller particle sizes showed weak Pd diffraction peaks, which indicated that ZnCl_2_ activated supports could prevent the aggregation of Pd particles.

**Figure 2:**
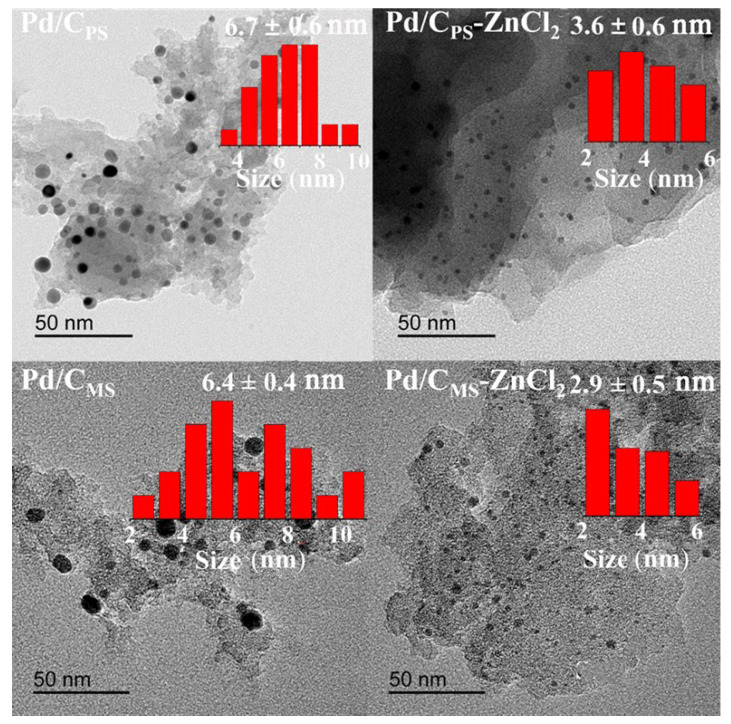
TEM micrographs and particle size distribution of various catalysts.

The dispersion of Pd particles was further observed by TEM. As displayed in Figure 2, Pd/C_PS_ and Pd/C_MS_ had large particle sizes and wide distributions. Pd/C_PS_-ZnCl_2_ and Pd/C_MS_-ZnCl_2_ had uniformly dispersed nanoparticles with average particle sizes of 3.6 and 2.9 nm, which was consistent with the results obtained by XRD. Therefore, ZnCl_2_ activation had a great influence on the particle size of Pd, which might be related to the structure and properties of the support.

**Figure 3:**
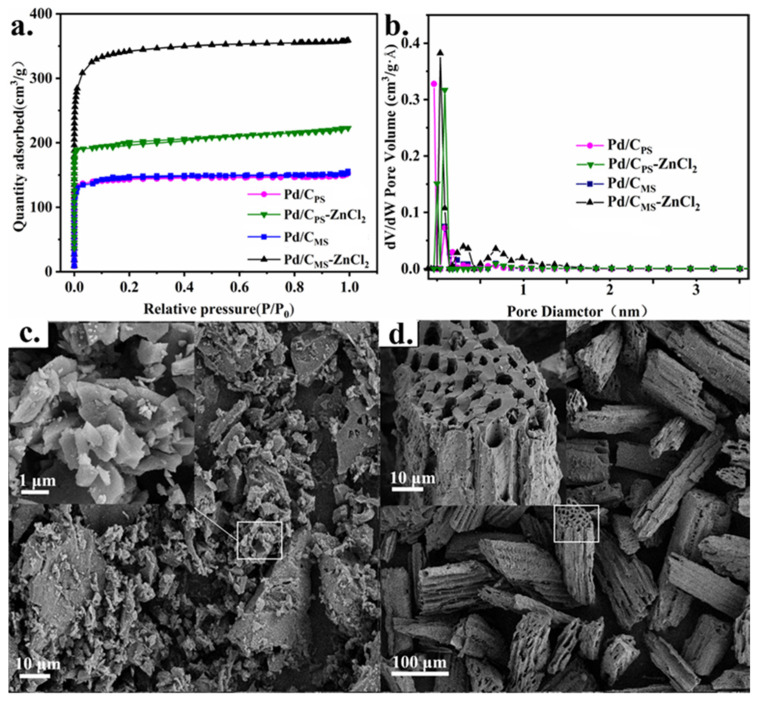
(**a**) N_2_ absorption–desorption isotherms and (**b**) pore sizes distribution of various catalysts. (**c**) SEM images of Pd/C_PS_-ZnCl_2_ and (**d**) Pd/C_MS_-ZnCl_2_.

Figure 3a,b demonstrated the N_2_ adsorption–desorption isotherms and the corresponding pore diameter distribution curves of the catalysts, and the corresponding calculation results are listed in Table 2. Type I isotherms in all catalysts confirmed microporous structure. Through the activation of ZnCl_2_, the specific surface area of Pd/C_PS_-ZnCl_2_ increased from 456 to 629 m^2^·g^−1^. Moreover, the surface area of Pd/C_MS_-ZnCl_2_ was 1081 m^2^·g^−1^, which was about 3-fold larger than that of Pd/C_MS_ (466 m^2^·g^−1^). It is rational that ZnCl_2_ reacted with O_2_ to form ZnO and Cl_2_, and the produced ZnO can further react with C atoms to generate CO_2_ and Cl_2_ in order to achieve the purpose of pore making [25,38]. When P/P_0_ was close to one, the adsorption capacity of all catalysts tended to be saturated and had no obvious hysteresis loop, which indicated that they had relatively small micropores. It can be observed from Figure 3b that the pore size of the catalysts was mainly distributed between 0.34 and 0.55 nm. Whether melon seed shells or peanut shells were used as precursor, the microporous structure of the catalysts activated by ZnCl_2_ was more abundant. Moreover, the micropore volume of Pd/C_MS_-ZnCl_2_ increased significantly from 0.20 to 0.44 cm^2^·g^−1^, which might depend on the nature of the biomass itself. The morphologies of Pd/C_PS_-ZnCl_2_ and Pd/C_MS_-ZnCl_2_ were characterized by SEM. In contrast to the disordered porous carbon obtained by carbonization of peanut seed shell (Figure 3c), the structure of melon shell presented a uniform cylindrical honeycomb (Figure 3d).

In addition to the structure and morphology analyzed in the previous discussion, the surface properties of catalysts are also keys for determining its catalytic performance. The isoelectric point (IEP) test showed that Pd/C_PS_ and Pd/C_MS_ had similar isoelectric points, which were 5.4 and 5.5 respectively. After activation, the isoelectric points of Pd/C_PS_-ZnCl_2_ and Pd/C_MS_-ZnCl_2_ increased to 6.5 and 6.9. From the reaction mechanism, FA is adsorbed on the Pd surface through deprotonation to form HCOO^−^. Therefore, high H^+^ concentration is harmful because too many H^+^ may promote the recombination of HCOO^−^ with H^+^ and/or delay the deprotonation step, resulting in the reduction in FA dehydrogenation rate [39].

**Figure 4:**
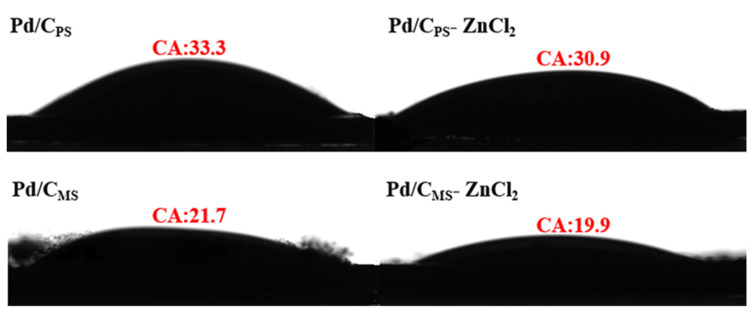
Photographs of water droplets on various catalysts thin film together with their contact angles.

It could be seen from the photographs of water droplets on the catalyst thin films in Figure 4 that the contact angles of Pd/C_PS_, Pd/C_PS_-ZnCl_2,_ Pd/C_MS_ and Pd/C_MS_-ZnCl_2_ were 33.3°, 30.9°, 21.7° and 19.9°, respectively. Pd/C_MS_, Pd/C_MS_-ZnCl_2_ obtained by carbonization of melon seed shell had higher hydrophilicity. Solid catalysts with strong hydrophilicity can improve the interface and enhance the performance of heterogeneous catalysts [40]. FTIR spectra analysis exhibited that the catalysts had absorption peaks at 3427.1 and 3427.7 cm^−1^ corresponding to the presence of an O-H group, and the peak at 1119.2 and 1123.3 cm^−1^ could be ascribed to a C-OH group (Figure S2). Due to the breaking and recombination of chemical bonds during biomass pyrolysis, a large number of oxygen-containing functional groups were formed on the surface of biomass carbon, which was also the main feature of biomass carbon that was different from other carbons [41].

**Figure 5:**
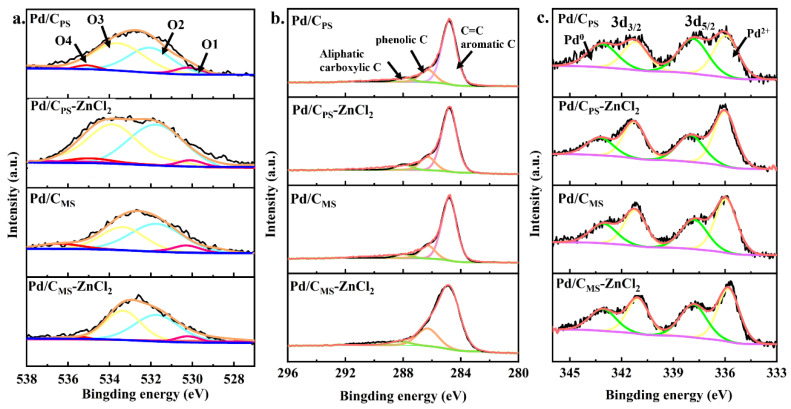
XPS patterns of (**a**) O 1s, (**b**) C 1s and (**c**) Pd 3d of various catalysts.

XPS spectra were used to analyze surface composition and chemical state of the catalysts. Figure 5a presented that the O 1s XPS spectra could be divided into four types of oxygen-containing functional groups: O1 (quinones, 530.8–531.3 eV), O2 (ethers or phenolic hydroxyl, 532.4–532.8 eV), O3 (lactone or carboxyl, 533.4–534.0 eV) and O4 (adsorbed O_2_ or H_2_O, 535.0–535.8 eV) [42], and the corresponding atomic ratios were listed in Table S1. The surface O content of and Pd/C_PS_-ZnCl_2_ and Pd/C_MS_-ZnCl_2_ increased by 63% and 55%, respectively, after ZnCl_2_ activation. Previous studies have showed that the oxygen-containing functional group on the surface of the support can be used as the nucleation point of metal particles to promote the interaction between the support and metal particles so as to form smaller nanoparticles [43,44]. Compared with Pd/C_MS_ (7.33 at%), activated Pd/C_MS_-ZnCl_2_ had more O2 and O3 contents (11.71 at%). At the same time, similar results were observed in the other group. The increase in O2 and O3 might increase the hydrophilicity of the catalyst to a certain extent [45]. From C 1s spectra (Figure 5b), the content of aromatic C (284.8 eV) [46] in all samples accounts for about 70%–80% of the total C content (Table S2). It was found that ZnCl_2_ activation reduced the content of aromatic C. In addition, phenolic C (286.4 eV) and aliphatic and carboxylic C (287.8 eV) were observed on the surface of catalysts. The ashes were obtained by calcining the sample at 650 °C for 2 h. The results revealed that the carbonization of peanut shell produced more ash than melon seed shell, and the ash content would decrease with the introduction of ZnCl_2_. In the XPS spectra of Pd 3d 5/2 (Figure 5c), two peaks were fitted well at 335.8 and 337.8 eV, relating to Pd^0^ and Pd^2+^, respectively. As observed in Table S3, there was no significant difference in the electronic state of Pd between different catalysts. Moreover, the Pd content of all catalysts was similar, both from the surface content determined by XPS and the bulk content determined by ICP.

### 3.2. Catalytic Activity

**Figure 6:**
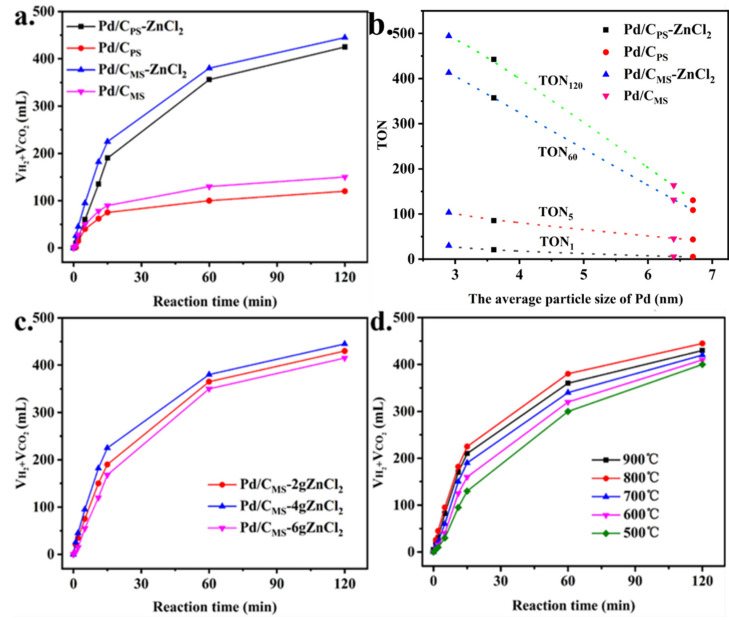
Total volume (H_2_ + CO_2_) generation catalyzed by (**a**) various catalysts, (**c**) Pd/C_MS_ with different ZnCl_2_ masses activated and (**d**) Pd/C_MS_-ZnCl_2_ with different carbonization temperature from FA. (**b**) Various catalysts with corresponding TON value vs. particle size of various catalysts.

The performance of the catalyst with different biomass supports the total volume (H_2_ + CO_2_) production at 30 °C as presented in Figure 6a. The best catalytic activity was observed for Pd/C_MS_-ZnCl_2_ in which the reaction was completed in 120 min, providing TON_120_ with a value of 484.3 (Table S4). The generated gas was determined by GC to be H_2_ and CO_2_ with the molar ratio of 1:1, where CO was below the detection limit (<1 ppm) (Figures S3 and S4). Similarly, Pd/C_PS_-ZnCl_2_ showed the second-best catalytic performance, with a TON_120_ value of 462.5 and conversion of 95% at 120 min. However, under the same conditions, the TON_120_ values of Pd/C_MS_ and Pd/C_PS_ were only 168.5 and 130.6, and the corresponding conversion rates were 34% and 27%, respectively. The relationship between specific catalytic activity expressed in TON and Pd particle size is observed in Figure 6b. The size of Pd particles was negatively correlated with the TON value of the catalysts, mainly because the Pd with smaller particle size had more active sites [47,48]. Moreover, the smaller nanoparticles had a “clean” surface (uncapped) and more active edge/corner atoms, so they showed excellent catalytic performance [13,49].

Through the characterization and performance evaluation of the catalysts, the supports played an important role in the activity of the catalysts. During the preparation of the supports, ZnCl_2_ had the functions of swelling, catalytic dehydration and pore forming in the treatment of biomass raw materials [50]. Therefore, the action of ZnCl_2_ activating biomass to obtain biomass carbon support with large specific surface areas was of great significance in reducing metal particles size and improving dispersion, as it increased catalyst activity. In addition, more oxygen-containing functional groups could be obtained through ZnCl_2_ activation, which could boost the dispersion of Pd nanoparticles and reduced surface acidity for the purpose of improving catalytic performance. Another important factor affecting the structure and surface properties of the supports depends on the properties of the raw material. Compared with peanut shells, the catalyst prepared by carbonizing melon seed shell had larger specific surface areas, higher hydrophilicity and uniform honeycomb morphology. Therefore, the selection of appropriate biomass precursors was also key to the preparation of high-performance catalysts.

The activity of catalysts prepared by activation of ZnCl_2_ with different masses on the decomposition of FA is observed in Figure 6c. The TON_1_ values of Pd/C_MS_-2gZnCl_2_ and Pd/C_MS_-6gZnCl_2_ catalyzed FA decomposition were 12.8 and 5.4. By comparison, the TON_1_ value of Pd/C_MS_-4gZnCl_2_ was 2-fold and 5-fold higher than those of Pd/C_MS_-2gZnCl_2_ and Pd/C_MS_-6gZnCl_2_, respectively. Therefore, it could be inferred that the quality of ZnCl_2_ affects activation efficiency. A suitable amount of ZnCl_2_ could adjust the porous structure of biomass carbons; in contrast, excessive ZnCl_2_ was likely to boost the volatilization of surface species of the support so as to widen the pores and reduce the surface area of biomass carbons [51].

Biomass carbon was mainly composed of aromatic carbon, amorphous carbon and ash, and the content of each part was mainly determined by pyrolysis temperature [52]. The swelling function of ZnCl_2_ and the release of small molecular gases could catalyze the aromatization reaction at temperature ≥ 400 °C and generate a large number of pore structures [36]. Therefore, the effect of carbonization temperature on catalyst activity was investigated from 500 °C (Figure 6d). When the carbonization temperature was 500 °C, the TON_1_ value of the catalyst was only 4.4. With the increase in carbonization temperature, the catalytic activity of the prepared catalyst gradually increased, and the TON_1_ value of 28.3 was the largest at 800 °C. This might be because at lower temperatures, water, CO and CO_2_ in biomass volatilize to form amorphous carbon. With the increase in temperature, alkyl carbon and oxyalkyl carbon fractured in biomass and gradually rearranged into aryl carbon [53,54]. Among them, aromatic hydrocarbon carbon has strong stability, and this structure of biomass carbon was also an important reason for its stability. However, the TON_1_ value of Pd/C_MS_-900 was 14.1. From the previous discussion, it could be seen that the surface oxygen-containing functional groups could promote the activity of the catalyst, while O content decreased with the increase in temperature, which might be the reason for decreased catalytic activity with the continuous increase in carbonization temperature.

**Figure 7:**
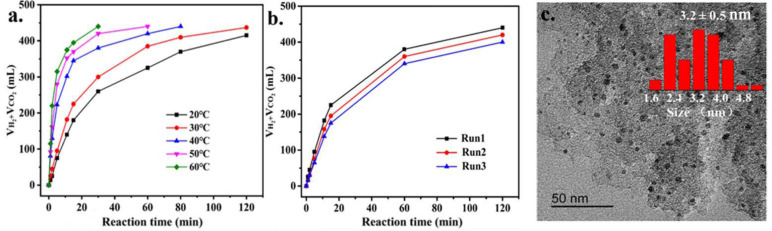
(**a**) Total volume (H_2_ + CO_2_) generation catalyzed by Pd/C_MS_-ZnCl_2_ at different reaction temperatures and (**b**) stability test for the decomposition for FA. (**c**) TEM micrographs and particle size distribution of the recycled Pd/C_MS_-ZnCl_2_.

The activity of Pd/C_MS-_ZnCl_2_ for FA decomposition at 20–60 °C was investigated in Figure 7a. With the increase in reaction temperature, the gas generation rate increased obviously, providing the TON_1_ values of 15.0, 28.3, 42.5, 63.1 and 100.1. The stability of the catalyst was important for practical applications. After three cycles, catalytic activity decreased to a certain extent (Figure 7b). TEM characterization of the catalyst after the reusability test found that the average particle size increased from the initial 2.9 nm (Figure 2) to 3.2 nm (Figure 7c). XPS characterization of the catalyst after the reaction demonstrated that the content of Pd^0^ decreased (Figure S5). In the dehydrogenation of formic acid, Pd^0^ is conducive to the activation of C-H bond, which is often the rate controlling step of dehydrogenation [50,55,56]. Therefore, the decrease in catalyst Pd^0^ in the process might also be another reason for the decline of activity.

## 4. Conclusions

To sum up, high efficiency Pd nanoparticles were loaded on peanut shell and melon seed shell derived porous carbon for FA dehydrogenation. The catalysts obtained from biomass activated by ZnCl_2_ had Pd particle size distributions of 2.9 nm (Pd/C_MS_-ZnCl_2_) and 3.6 nm (Pd/C_PS_-ZnCl_2_), which were smaller than those of the unactivated catalyst. The analysis showed that the specific surface area of the catalysts was improved by ZnCl_2_ treatment; Pd/C_MS_-ZnCl_2_, in particular, had a high specific surface area of 1081 m^2^·g^−1^. In addition, ZnCl_2_ also affected the surface properties of catalysts. The surface O content of Pd/C_PS_-ZnCl_2_ and Pd/C_MS_-ZnCl_2_ increased by 63% and 55%, respectively. Moreover, the activated catalysts were closer to neutral, which would be conducive to the removal of H^+^ from FA. The properties of biomass also determined the performance of the catalyst. Notably, Pd/C_MS_-ZnCl_2_ afforded the highest TON_1_ value, reaching up to 28.3 at 30 °C, which is higher than that of Pd/C_PS_-ZnCl_2_ (19.0). The Pd/C_MS_-ZnCl_2_ with melon seed shells as precursor had higher specific surface area; thus, it could provide more sites for Pd nanoparticles. Moreover, its homogeneous honeycomb structure and stronger hydrophilicity further promoted the decomposition rate of liquid FA. This study not only provides a method for using agricultural waste as a carbon source of porous carbon but also accelerates FA as a high-efficiency hydrogen supplier in mobile devices.

## Figures and Tables

**Table 1:** Pd particle sizes of various catalysts.

Catalyst	Average Particle Size (nm)	
	XRD	TEM
Pd/C_PS_	7.2	6.7 ± 0.6
Pd/C_PS_-ZnCl_2_	3.8	3.6 ± 0.6
Pd/C_MS_	6.8	6.4 ± 0.4
Pd/C_MS_-ZnCl_2_	2.8	2.9 ± 0.5

**Table 2:** Textural properties of various catalysts.

Catalyst	SSA_BET_ ^1^ (m^2^·g^−1^)	SSA_Mes_ ^2^ (m^2^·g^−1^)	SSA_Mic_ ^3^ (m^2^·g^−1^)	V_T_ ^4^ (cm^3^·g^−1^)	V_Mes_ ^5^ (cm^3^·g^−1^)	V_Mic_ ^6^ (cm^3^·g^−1^)
Pd/C_PS_	456	50	406	0.23	0.03	0.20
Pd/C_PS_-ZnCl_2_	629	113	517	0.34	0.09	0.25
Pd/C_MS_	466	50	416	0.24	0.04	0.20
Pd/C_MS_-ZnCl_2_	1081	199	882	0.55	0.11	0.44

^1^ Specific surface area, ^2^ mesopore and ^3^ micropore specific surface area. ^4^ Total pore volume, ^5^ mesopore and ^6^ micropore pore volume.

## Data Availability

Not applicable.

## Supplementary Materials

The following are available online at https://www.mdpi.com/article/10.3390/nano11113028/s1, Figure S1: Experimental apparatus of H_2_ generation from the FA dehydrogenation, Equation (S1): The average particles sizes were estimated from XRD by Debye-Scherrer, Figure S2: The spectrum of various catalysts, Figure S3: GC spectrum using TCD for the gas from FA over Pd/C_MS_-ZnCl_2_, Figure S4: GC spectrum using PDHID for the standard gas and the gas from FA over Pd/C_MS_-ZnCl_2_, Figure S5: XPS patterns of Pd 3d of Pd/C_MS_-ZnCl_2_ before and after reaction, Table S1: O content of various catalysts (at.%), Table S2: C content and Ash of various catalysts, Table S3: Pd content of various catalysts, Table S4: FA dehydrogenation catalyzed by various catalysts.
