# Supplementary material for: Facile Synthesis of Microporous Carbons from Biomass Waste as High Performance Supports for Dehydrogenation of Formic Acid
Source: Nanomaterials (Basel). 2021 Nov 11;11(11):3028. doi: 10.3390/nano11113028 (PMC8624553; doi:10.3390/nano11113028)
Supplement: Supplementary file 1 [file nanomaterials-11-03028-s001.zip › nanomaterials-1444780-supplementary.pdf]

nanomaterials

Supplementary Materials

Facile synthesis of microporous carbons from biomass waste as high performance supports for dehydrogenation of formic acid

Tingting Cao [1,2,†], Jinke Cheng [1,2,†], Jun Ma [1,2], Chunliang Yang [1,2], Mengqin Yao [1,2,*], Fei Liu [1,2,*], Min Deng [1,2], Xiaodan Wang [1,2] and Yuan Ren [2,*]

[1] School of Chemistry and Chemical Engineering, Guizhou University, Guiyang 550025, China; caotingting980417@163.com (T.C.); jkcheng@gzu.edu.cn (J.C.); jma3@gzu.edu.cn (J.M.); clyang@gzu.edu.cn (C.Y.); gzu403299473@163.com (M.D.); wangxiaodan0516@126.com (X.W.)

[2] Key Laboratory of Green Chemical and Clean Energy Technology, Guizhou University, Guiyang 550025, China

* Correspondence: mqyao@gzu.edu.cn (M.Y.); ce.feiliu@gzu.edu.cn (F.L.); renyuan_1986@126.com (Y.R.)

† These two authors contributed equally to this work.

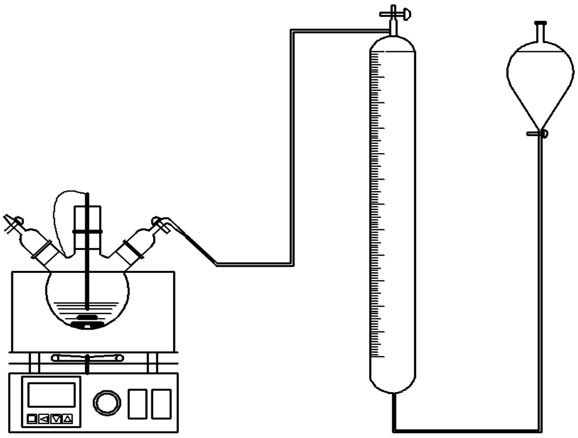

Figure S1. Experimental apparatus of H_2 generation from the FA dehydrogenation.

The average particles sizes were estimated from XRD by Debye-Scherrer. Equation (S1):

$$D = {}^{K\lambda}/_{BCOS\theta} \quad (1)$$

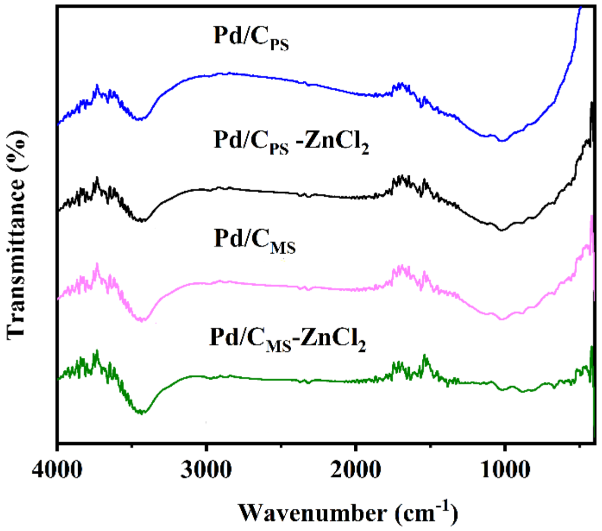

Figure S2. The spectrum of various catalysts.

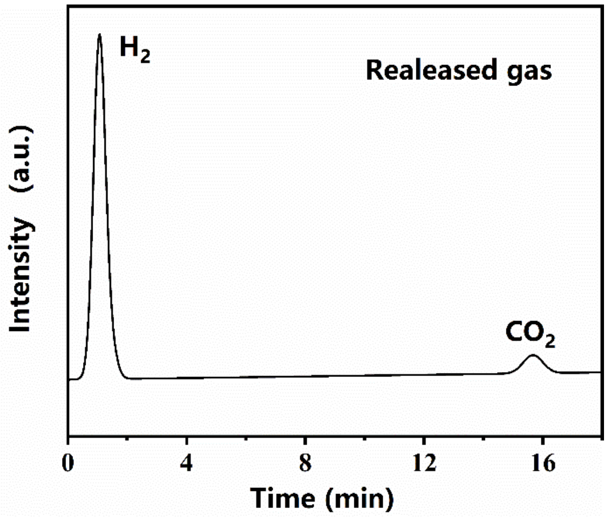

Figure S3. GC spectrum using TCD for the gas from FA over Pd/C_{MS}-$ZnCl_2$.

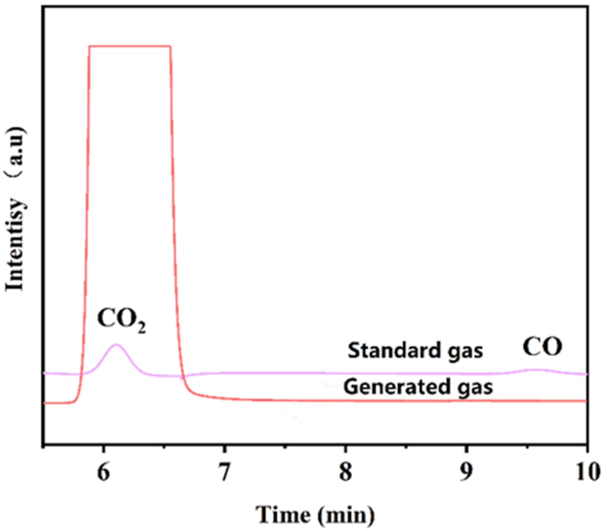

Figure S4. GC spectrum using PDHID for the standard gas and the gas from FA over Pd/C_{MS}-$ZnCl_2$.

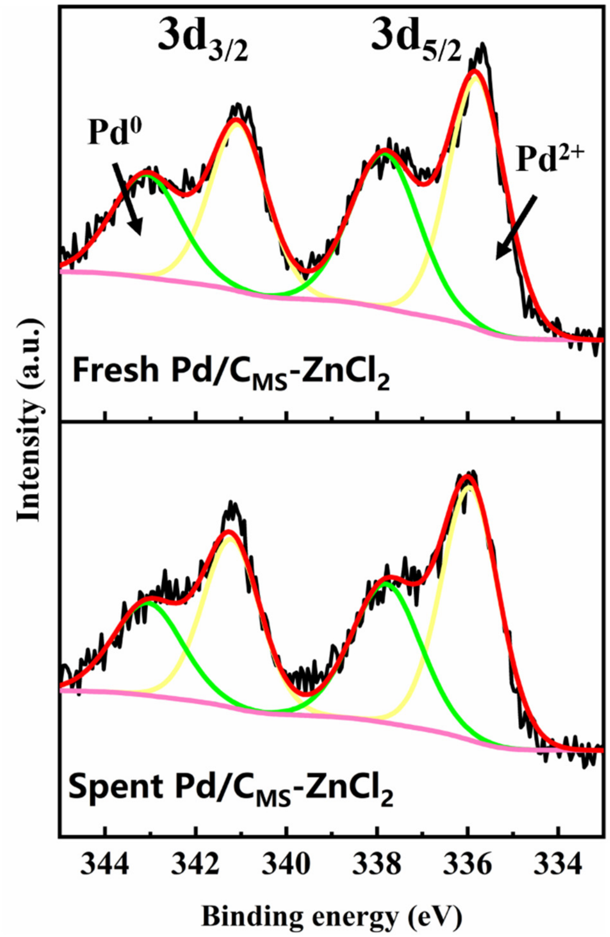

Figure S5. XPS patterns of Pd 3d of Pd/C_{MS}-$ZnCl_2$ before and after reaction.

Table S1. O content of various catalysts (at %).

Catalysts	O	O1	O2	O3	O4
Pd/C_{PS}	6.30	0.36	2.31	3.30	0.33
Pd/C_{PS}-$ZnCl_2$	10.26	0.40	4.92	4.44	0.50
Pd/C_{MS}	8.06	0.29	3.03	4.30	0.44
Pd/C_{MS}-$ZnCl_2$	12.50	0.44	5.98	5.73	0.35

Table S2. C content and Ash of various catalysts.

Catalysts	XPS (at.%)				Ash (wt%)
	C	C=C aromatic C	phenolic C	aliphatic carboxylic C	
Pd/C_{PS}	89.17	70.19	14.86	4.12	4.38
Pd/C_{PS}-$ZnCl_2$	85.10	67.67	13.84	3.59	3.19
Pd/C_{MS}	87.47	69.76	14.08	3.63	0.60
Pd/C_{MS}-$ZnCl_2$	83.00	61.74	17.34	3.92	0.21

Table S3. Pd content of various catalysts.

Catalysts	XPS (at.%)			ICP (wt%)
	Pd	Pd^0	Pd^{2+}	Pd
Pd/C_{PS}	4.53	2.99	1.54	4.76
Pd/C_{PS}-$ZnCl_2$	4.64	2.96	1.68	4.78
Pd/C_{MS}	4.47	2.91	1.56	4.67
Pd/C_{MS}-$ZnCl_2$	4.50	2.90	1.60	4.70

Table S4. FA dehydrogenation catalyzed by various catalysts.

Catalysts	TON_X			
	1	5	60	120
Pd/C_{PS}	5.3	43.5	108.8	130.6
Pd/C_{PS}-$ZnCl_2$	19.0	92.3	387.4	462.5
Pd/C_{MS}	5.6	54.4	141.5	168.5
Pd/C_{MS}-$ZnCl_2$	28.3	103.3	413.5	484.3
